# Decoding Bitter Taste Perception in Medicine-Food Homology Substances: A Multimethod Data and Sensory Assessment Approach

**DOI:** 10.3390/foods15183181

**Published:** 2026-09-09

**Authors:** Zhu Tian, Ji Wang, Shi-Fang Wu, Xu-Hui Huang, Lei Qin, Wei Liang

**Affiliations:** 1 State Key Laboratory of Marine Food Processing & Safety Control, School of Food Science and Technology, National Engineering Research Center of Seafood, Dalian Polytechnic University, Dalian 116034, China; 2 College of Biological Science and Technology, Yili Normal University, Yining 835000, China

**Keywords:** medicine-food homology (MFH), bitterness, brain perception, scalp electroencephalogram, taste, multimethod

## Abstract

Medicine and Food Homology (MFH) substances are often limited by bitter off-flavors that may limit consumer acceptance. This study employed a multimethod approach integrating electronic nose (E-nose), sensory evaluation, electronic tongue (E-tongue), gas chromatography-ion mobility spectrometry (GC-IMS), and electroencephalography (EEG) to investigate bitterness perception in five representative MFH extracts (Chenpi, Lotus leaf, Sichuan pepper, Gardenia, and American ginseng). Sensory and E-tongue analyses revealed discrepancies between perceived and chemically measured bitterness. GC-IMS identified distinct volatile fingerprints, highlighting differences in aroma composition among samples. EEG results showed that several conventionally bitter extracts were primarily associated with low-frequency scalp-EEG activity (δ–α bands), whereas Sichuan pepper showed an apparent delayed broadband increase extending into the β–γ range. Descriptive scalp EEG patterns varied across MFH stimulation conditions, including differences at frontal and temporal electrodes. Overall, this study provides an exploratory multimethod characterization of the sensory, instrumental, and scalp-EEG responses elicited by different MFH extracts.

## 1. Introduction

Medicine and Food Homology (MFH) ingredients have gained growing attention and have become a prevailing trend in modern dietary habits and healthcare practices. MFH substances not only provide nutritional value but also contribute to health promotion, providing functional benefits beyond those of conventional foods [1,2,3]. Currently, 106 distinct types of MFH ingredients are officially recognized by the National Health Commission of China and the State Administration for Market Regulation. However, numerous MFH materials possess undesirable taste profiles, especially bitterness, which severely impairs their palatability [4]. Bitter compounds are prevalent in MFH, primarily originating from plant metabolic processes. Plant metabolites, including alkaloids, polyphenols, amino acids, flavonoids, and terpenoids, are typically associated with a distinct bitterness [5].

Conventional flavor assessment primarily depends on sensory assessment, employing explicit metrics like sensory scores to define taste characteristics. For instance, Yang et al. [6] combined instrumental analysis with sensory tests to identify 15 key bitter compounds in Zanthoxylum bungeanum Maxim. Nonetheless, sensory approaches are constrained by individual variability in taste sensitivity, cultural preferences, and semantic changes, undermining their objectivity and reproducibility. These constraints highlight the demand for neurophysiological tools to compensate for the subjectivity of conventional sensory evaluation [7].

In recent years, biomimetic sensing systems, including electronic nose (E-nose) and electronic tongue (E-tongue), have been widely utilized in food flavor analysis. These systems utilize chemical sensor methods to mitigate perceptual bias, facilitating comprehensive characterization of flavor profiles, including various bitter off-flavors. They produce consistent and impartial data to facilitate studies on off-flavor development. Although basic qualitative and quantitative descriptions are offered, sensory evaluation and biomimetic sensing remain inadequate in fully elucidating the intricacies of bitter off-flavors. Instrumental approaches lack the capacity to reflect subjective hedonic perception, restricting comprehensive understanding of taste cognition and emotional responses [8]. This limitation is particularly prominent when analyzing MFH materials such as Gardenia and Sichuan pepper.

Researchers have utilized electroencephalography (EEG) to assess consumer sensory experiences, effectively addressing various challenges in this field. EEG is a non-invasive method for measuring brain activity, which provides distinct advantages in taste perception studies. Its exceptional temporal resolution allows for the recording of instantaneous neural responses, making it particularly valuable for this type of research [9]. It has been widely applied to investigate basic tastes, sweet, sour, bitter, and salty, revealing the temporal dynamics of cortical responses evoked by different tasting stimuli [8,10]. Wu et al. [11] found that disodium succinate, monosodium glutamate, and disodium inosine monophosphate could be discriminated by EEG analysis on a time scale of 5−6 s. Furthermore, umami stimuli significantly enhance the α wave activity, with the strongest response at 10 Hz, particularly in the frontal, parietal and occipital regions (*p* < 0.001). Nevertheless, comprehensive investigations into the EEG features and perceptual mechanisms that underlie the distinct bitterness of MFH constituents remain insufficient. Integrating EEG into MFH flavor research can provide new insights into the central processing of bitterness off-flavors and their cortical representations. Furthermore, an objective assessment of bitter off-flavor intensity in MFH materials can offer theoretical guidance for off-flavor mitigation and product quality improvement.

Sensory evaluation characterizes perceived attributes, whereas E-nose, E-tongue, and GC-IMS provide complementary instrumental information on volatile and taste-related response patterns. EEG data provide both an implicit and objective metric to investigate the unique brain responses elicited by different intensities of bitter off-flavors in MFH constituents. The comparative study of EEG findings across varying levels of bitter off-flavor provides novel insights into taste perception and underscores the distinct sensory characteristics of MFH’s bitter off-flavor. Therefore, this study intends to explore the bitter off-flavor attributes of MFH through sensory evaluation, E-tongue, E-nose, GC-IMS, and EEG analysis while also evaluating the EEG application in sensory assessment and providing innovative insights for improving sensory attributes and promoting the commercial utilization of MFH with unpleasant flavor profiles.

## 2. Materials and Methods

### 2.1. Materials

Five MFH samples were selected to represent different samples of bitter off-flavor, including Chenpi, Lotus leaf, Sichuan pepper, Gardenia, and American ginseng, obtained from Sinopharm Group Guizhou Great Health Industry Development Co., Ltd. (Guizhou, China). Detailed information regarding the botanical materials is provided in Table S1. These materials were selected to provide contrasting MFH stimulation conditions rather than chemically or perceptually equivalent bitterness stimuli.

### 2.2. Sample Preparation

To ensure that each raw material adequately reflects perceptual differences, a preliminary experiment was first conducted to screen and determine an optimal concentration. Extracts were initially prepared at 1%, 3%, 5%, 7%, and 10% (*w*/*v*). Each concentration was evaluated by 12 trained assessors for bitterness intensity, overall flavor intensity, oral irritation, and acceptability. A concentration was considered suitable when the bitterness and characteristic flavor were clearly perceptible, while excessive bitterness, astringency, or irritation that could interfere with repeated evaluation was avoided. Based on these criteria, 5% (*w*/*v*) was selected.

For formal experiments, each extract was prepared at 5% (*w*/*v*) by adding 5.0 g of the corresponding dried and ground plant material to 100 mL of purified water. The mixture was maintained at 95 °C for 30 min and subsequently ultrasonicated for 30 min at 40 kHz and 300 W. After extraction, the samples were filtered three times through 6 gauze layers. The residue and extraction vessel were rinsed with purified water, and the filtrate was adjusted to a final volume of 100 mL. The extracts were cooled to room temperature before analysis. The pH, total soluble solids, and turbidity of each extract were measured using a calibrated pH meter, a digital refractometer at 20 °C, and a turbidimeter, respectively. The results are summarized in Table S2.

### 2.3. Sensory Evaluation

Thirty participants (15 females and 15 males; age range: 18–30 years) were recruited from Dalian Polytechnic University. All participants were nonsmokers, had no self-reported taste or olfactory disorders, food allergies to the tested materials, neurological or psychiatric disorders, or current use of medications known to affect chemosensory perception. Participants were instructed to avoid food and beverages, except water, for at least 2 h and to avoid alcohol, tobacco, coffee, and strongly flavored foods for 4 h before each session.

Prior to the formal experiment, all candidate panelists underwent systematic screening following ISO 6658:2017 [12] sensory analysis procedures [8]. The screening included basic taste identification tests (sweet, sour, salty, bitter, umami). Only candidates with normal taste function who could correctly identify basic tastes and whose bitterness thresholds fell within the normal range were enrolled in the panel. Sensory assessment was also referred to as evaluation guideline for bitterness intensity of beverages/alcoholic beverages. All panelists received a briefing on the experimental protocol and gave informed consent before taking part. The Panel members were required to initially develop the ability to assess the bitterness intensity (0–10 points) of the samples by getting familiar with the bitter standard solutions of quinine hydrochloride at different concentrations through sensory perception in advance. A bitterness-intensity assessment was conducted with reference to T/CSTM 01249-2024 [13], using quinine hydrochloride solutions as reference stimuli.

All samples in the actual experiment were randomly marked with numbers to guarantee unbiased evaluation. To reduce the impact of personal biases, identical quantities of the same samples were supplied to the evaluators. Following the assessment, the evaluators rinsed their mouths with water, rested for 2 min, and subsequently proceeded to the next sample. The assessors were tasked with distinguishing the samples in each set based on their flavor characteristics and intensity, with each MFH sample assessed 3 times. Sensory characteristics (primarily bitterness) were rated on a scale from 0 (absent) to 10 (extremely strong).

### 2.4. E-Tongue Analysis

Samples of MFH extracts were examined based on the previous method [14], using an electronic tongue system (TS-5000Z, Intelligent Sensor Technology Inc., Atsugi, Japan) equipped with CA0, C00, AE1, AAE and CT0 lipid/polymer membrane sensors and the corresponding reference electrodes. These sensors were used to characterize relative responses associated with sourness, bitterness, astringency, umami, and saltiness. Moreover, 40 mL of MFH samples were added to the same volume of deionized water, and then mixed thoroughly and centrifuged for 15 min (4 °C, 6000 rpm). Then, the supernatant was gathered, and the filtrates were tested via E-tongue. Before measurement, the sensors were conditioned and calibrated in the manufacturer-specified reference solution. Each group of samples was performed in triplicate.

### 2.5. E-Nose Analysis

Samples of MFH extracts were prepared following a procedure described in a prior study [15]. The E-nose (PEN 3, Airsense Analytics GmbH, Schwerin, Germany) was equipped with ten metal-oxide semiconductor sensors: W1C, W5S, W3C, W6S, W5C, W1S, W1W, W2S, W2W, and W3S. These sensors corresponded to various volatile compounds. Each sensor could operate independently and be disassembled separately. Prior to testing, different extract samples underwent preincubation, with 5 mL of the extracts added to 20 mL airtight vials. The incubation temperature was maintained at 60 ± 2 °C, and the analysis duration was 120 s. Each group of samples was performed in triplicate.

### 2.6. GC-IMS Analysis

Analysis of volatiles in MFH samples referred to the method described by Liang et al. [16]. VOCs in MFH extracts were assessed through the GC-IMS (Flavorspec^®^, G.A.S., Dortmund, Germany) equipped with an autosampler and a DB-WAX capillary column (30 m × 0.32 mm × 0.25 µm). For each measurement, 3 mL samples were injected into the headspace vial (20 mL), sealed, and incubated for 15 min (60 °C). Subsequently, the determination was carried out based on the parameters of the previous laboratory.

High-purity nitrogen (≥99.999%) was used as both the carrier and drift gas. The carrier-gas flow program was as follows: 2 mL/min for 0–2 min, increased to 10 mL/min over 10 min, increased to 100 mL/min over 20 min, and then increased to 150 mL/min until the end of the run. The drift tube was maintained at 45 °C with a constant drift-gas flow of 150 mL/min. The total analysis time was 31 min.

Retention indices were calculated using an external series of n-ketones analyzed under the same chromatographic conditions. VOCs were tentatively identified by comparing both their experimental retention indices and normalized drift times with those in the GC-IMS library. Monomer and dimer signals were recorded separately when detected. Because compound identification was based on library matching rather than confirmation with authentic standards, the assignments are regarded as tentative.

Raw data were processed using VOCal software version 0.4.03. Reporter and Gallery Plot plug-ins were used to generate two-dimensional spectra and fingerprint plots. Each extract was analyzed using 3 independently prepared replicates. Peak volumes were normalized to the total signal intensity within each chromatogram.

### 2.7. EEG Participants

Thirty sensory panelists, comprising 15 males and 15 females from Dalian Polytechnic University, who previously took part in the sensory assessment, were enlisted for this research. They all possessed typical taste and sensory abilities, maintained robust physical and mental health, and had no record of neurological or psychiatric issues in themselves or their families. To reduce external influences, they were directed to steer clear of fragrant items for 6 h and abstain from consuming food, beverages, or tobacco for 2 h before the trial. Furthermore, participants were instructed to cleanse their hair to eliminate dust and oil from their scalps. The test was performed in a secure, tranquil, odorless EEG laboratory kept at a stable temperature of 21 ± 2 °C. Prior to the study, all participants provided written consent.

### 2.8. EEG Recording

EEG data were collected using the 32-channel ErgoLAB EEG system (Beijing Jinfa Technology Co., Ltd., Beijing, China) based on the international 10–20 system. Data were recorded at a sampling rate of 1000 Hz, with electrode impedance kept below 5 kΩ. Participants were directed to stay motionless and concentrate on taste perception to reduce motion artifacts [17].

### 2.9. Formal EEG Experiment

Participants donned electroencephalogram (EEG) hats to ensure a proper fit between the hats and their scalps and were instructed to maintain emotional stability throughout the experiment by taking deep breaths. Participants cleaned their mouths with pure water 3 times to create the 30 s baseline at the commencement of each session [8]. Following this, a personal assistant provided various MFH stimulus samples randomly, comprising water (control) and five distinct raw material solutions, each 1 ml in volume. Participants sampled the substances deliberately in accordance with the training directives, refraining from swallowing or moving their heads to reduce artifacts to the greatest extent possible.

**Figure 1:**
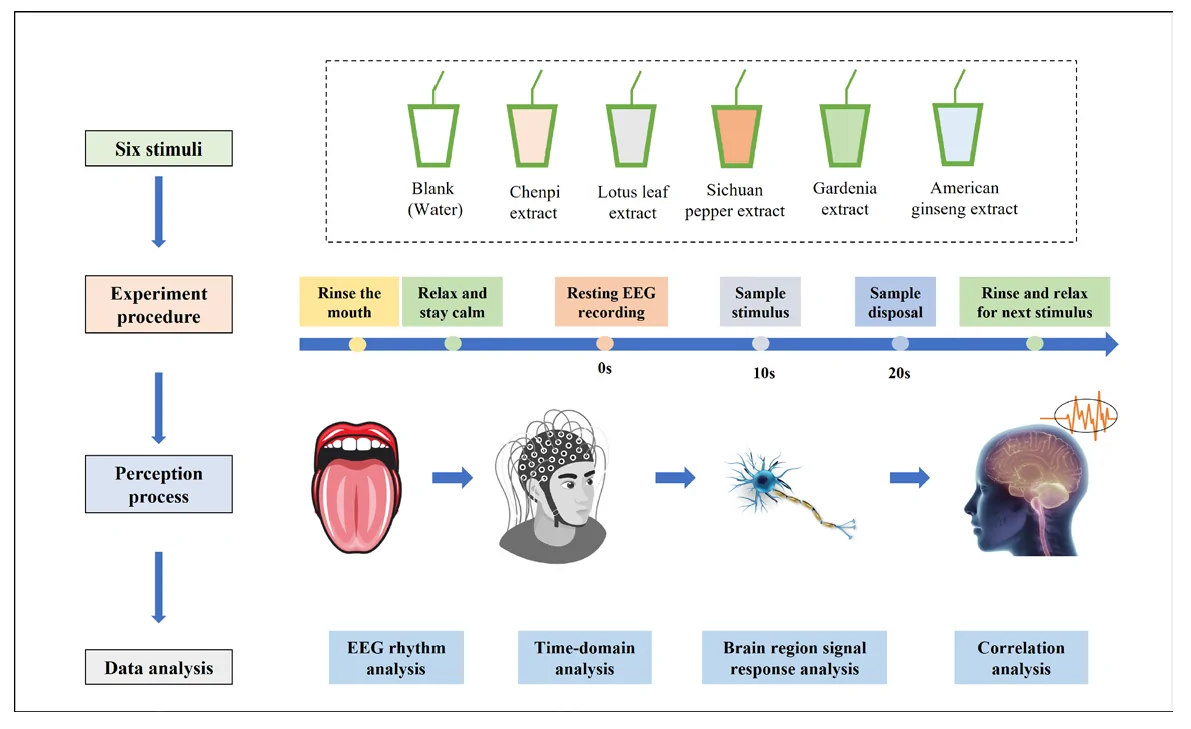
Schematic of the electroencephalography (EEG) experimental protocol.

Upon achieving a stable baseline, EEG recording commenced promptly upon the introduction of the stimulation solution into the subject’s oral cavity, with the initiation of data collection by pressing the recording button (as shown in Figure 1). Participants tasted the MFH stimulation samples slowly without swallowing or moving their heads, and spat them out after 10 s. Then, they rinsed their mouths with pure water 2 to 3 times to fully ensure the accuracy of the tasting and evaluation. In addition, they were required to take proper breaks to minimize the lingering aftertaste. Following a 120 s break, participants were directed to unwind and let the electric signal revert to its initial level. After the baseline stabilized, we documented it for 30 s without any stimulation, then proceeded with introducing the subsequent stimulus.

### 2.10. EEG Data Preprocessing and Analysis

The EEG data was subjected to preprocessing and feature extraction using ErgoLAB software (ErgoLAB 3.0). Re-referencing was conducted utilizing the average reference approach, with a filter bandwidth of 0.1–70 Hz. A 50 Hz notch filter was employed to mitigate power line interference.

**Figure 2:**
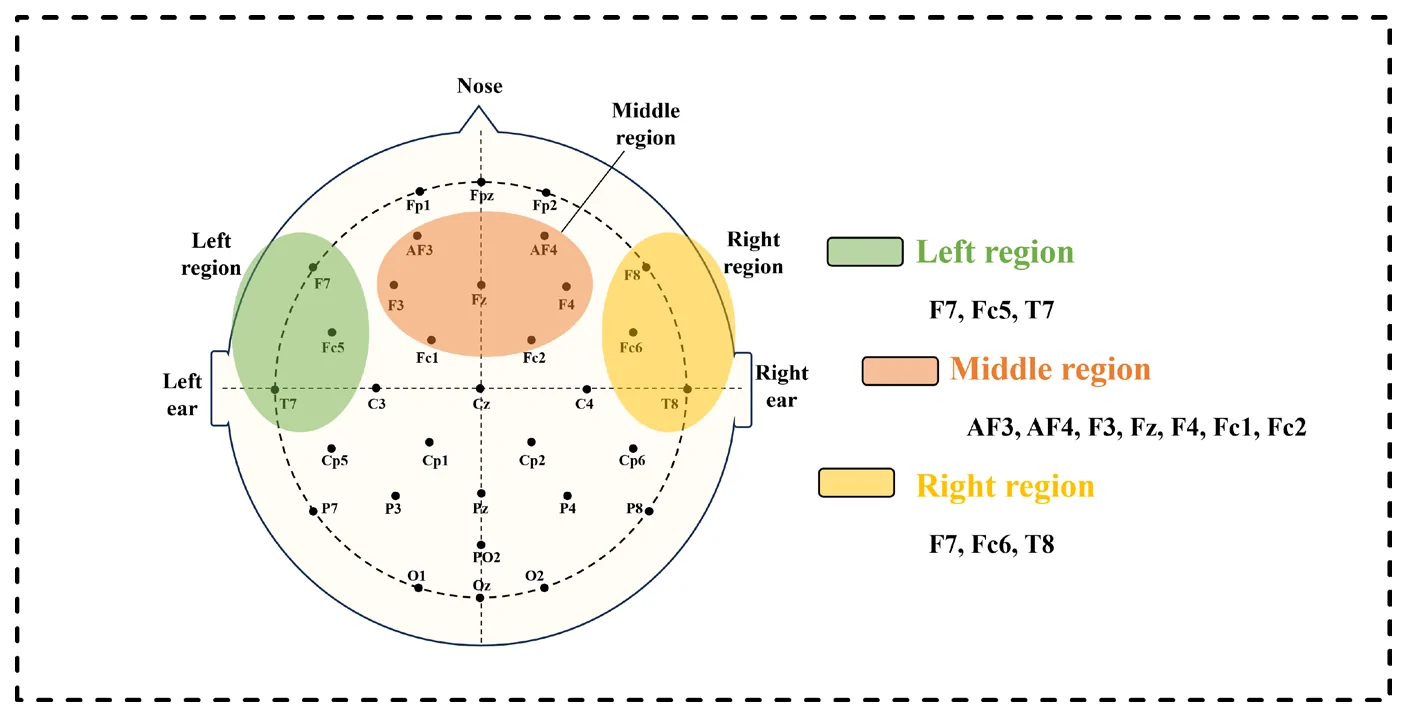
Region diagram of the brain structure: Left region for F7, Fc5, T7; Right region for F8, Fc6, T8; Middle region for AF3, AF4, F3, Fz, F4, Fc1, and Fc2.

Spectral characteristics were derived following the approach outlined by Gil Avila et al. [18]. The area under the curve (AUC) of the power spectral density (PSD) was computed for the δ, θ, α, β, and γ (respectively, 1–4 Hz, 4–8 Hz, 8–14 Hz, 14–30 Hz, 30–49 Hz) frequency bands during the tasting evaluation stage, in addition to each channel. To further investigate how taste perception from different MFH extracts affects brain signal responses, we focused on three specific brain regions. These regions were chosen based on prior research and the actual power distribution observed in this study. The signal analysis was divided into three segments: the left region, middle region, and right region (Figure 2). Various stimulus types, left, middle, and right regions, as well as delta (δ), theta (θ), alpha (α), beta (β), and gamma (γ) rhythm waves, were designated as fixed factors, with participants as random factors. The association between stimulus types and changes in brain activation was analyzed using the Pearson correlation test.

### 2.11. Scalp Topographical Visualization

Scalp potential distributions were visualized using the electrode-level EEG signals recorded by the 32-channel system. Topographical maps were generated by interpolating the measured scalp potentials across electrode locations during the selected post-stimulus interval. EEG signals collected during the tasting stage, spanning the first 10 s (0–10 s) after flavor stimulation, were averaged [8].

### 2.12. Statistical Analysis

Statistical analyses were performed using IBM SPSS Statistics 20.0. Data are presented as mean ± SD. Normality was assessed using the Shapiro–Wilk test and Q-Q plots. Repeated-measures ANOVA with Greenhouse-Geisser correction, where necessary, was used for sensory and EEG data, followed by Bonferroni-adjusted comparisons. Effect sizes were reported as partial eta squared (ηp^2^). PCA was used to visualize E-tongue and GC-IMS profiles. Pearson correlations were visualized using Origin 2026, with *p* values adjusted by the Benjamini–Hochberg procedure. All tests were two-tailed, with adjusted *p* < 0.05 considered significant.

## 3. Results and Discussion

### 3.1. Traditional Evaluation Approaches for Various MFH Extracts

**Figure 3:**
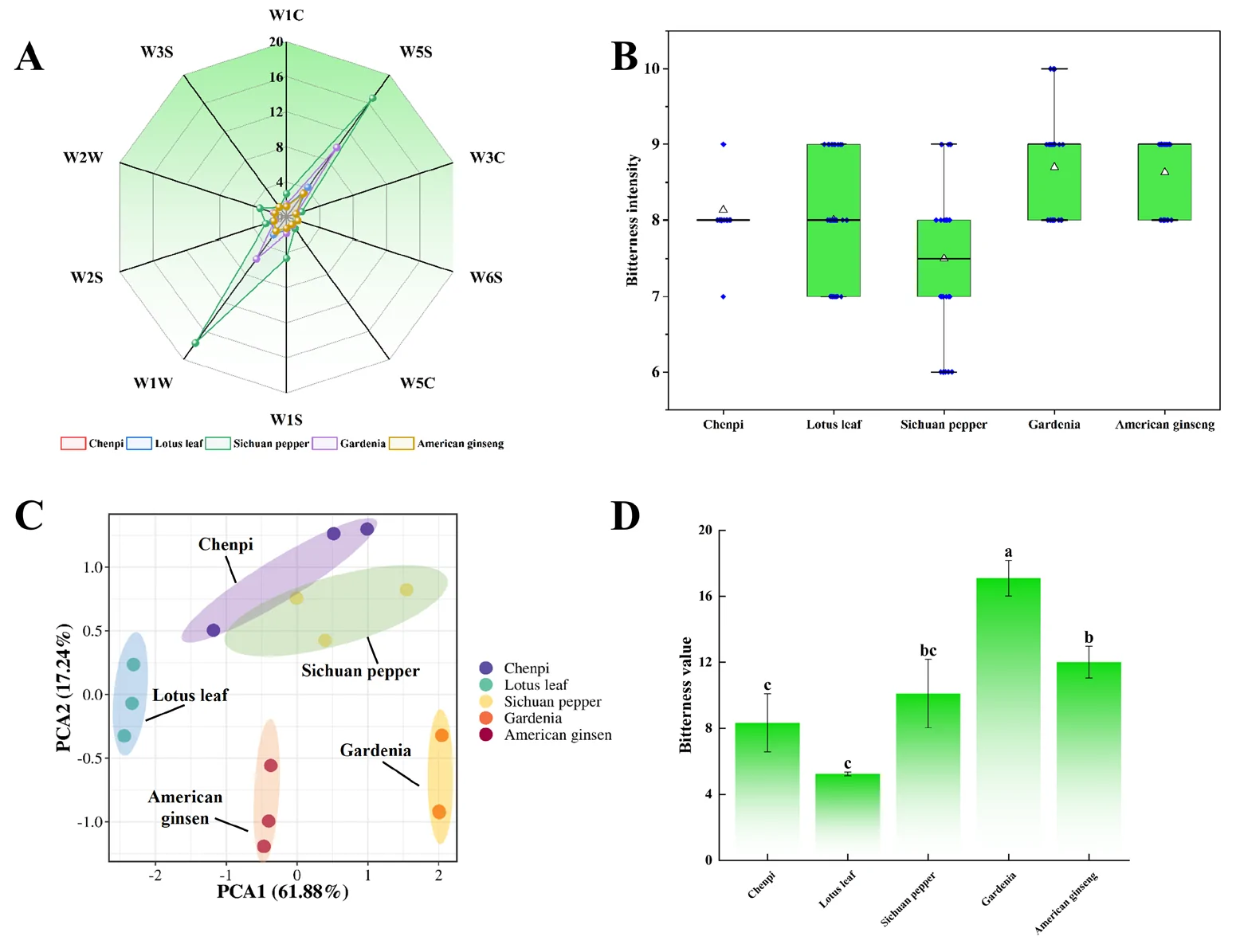
(**A**) Flavor differences among different MFH samples by E-nose (n = 3). (**B**) Sensory evaluation of different MFH samples (n = 30). Boxes represent the interquartile range, horizontal lines indicate medians, open triangles indicate means, whiskers extend to the most extreme values within 1.5 times the interquartile range, and blue points represent individual participant scores. (**C**) The PCA of different MFH samples by E-tongue (n = 3). (**D**) Bitterness value of different MFH samples by E-tongue (n = 3). Different lowercase letters indicate significant differences among MFH extracts (*p* < 0.05). Each point in (**C**) represents one analytical replicate.

To objectively characterize the flavor profiles, we first employed an E-nose, and then we illustrate the volatile response patterns with the E-nose radar chart (Figure 3A). Sensors W5S (sensitive to nitrogen oxides) and W1W (sensitive to sulfur compounds) demonstrated dominant responses across all MFH samples (especially the Sichuan pepper), while other sensors remained at baseline levels. Given the broad cross-sensitivity of E-nose sensors, these responses reflect differences in the overall volatile fingerprints of the samples [19]. Identification of individual VOCs was therefore based on the complementary GC-IMS analysis.

The sensory evaluation results for the five MFH extracts are presented in Figure 3B. Gardenia and American ginseng exhibited relatively high bitterness intensities, with median scores of approximately 9. Chenpi scores were concentrated around 8, whereas lotus leaf showed a broader distribution ranging mainly from 7 to 9. Sichuan pepper had the lowest median score (approximately 7.5) and the greatest inter-individual variability, with scores ranging from 6 to 9. This relatively lower perceived bitterness in Sichuan pepper may be attributed to the masking effect of hydroxy-α-sanshool, the key compound responsible for the “numbing” sensation, which can interfere with the brain’s processing of bitter signals [20]. However, hydroxy-α-sanshool was not quantified, and the present design did not isolate trigeminal stimulation; therefore, any masking or modulatory effect remains hypothetical.

The E-tongue analysis is not influenced by subjective psychological factors, environmental interference or personal preferences, which can provide standardized data and eliminate individual differences in manual evaluation [21]. In order to further verify the accuracy of sensory evaluation, we further analyzed the intensity of the E-tongue’s response to different MFH samples. The PCA of the taste data (Figure 3C) indicated distinct clustering for each MFH extract without overlap (PCA1 + PCA2 > 80%), confirming that each material possesses a unique taste fingerprint. Furthermore, we analyzed the bitterness values of different MFH samples via E-tongue data. As shown in Figure 3D, the Gardenia extract displayed the highest bitterness value, significantly surpassing all other groups (*p* < 0.05), followed by American ginseng. Interestingly, while Lotus leaf showed moderate bitterness in human sensory evaluation (Figure 3A), it registered the lowest bitterness intensity in the E-tongue analysis (Figure 3C). This discrepancy highlights that human bitterness perception is often multidimensional and influenced by aroma and texture [22]. Consequently, five different MFH extracts with varied flavor profiles were chosen for the EEG studies to investigate the neural processes related to these sensory distinctions.

### 3.2. GC-IMS Analysis of Different MFH Extracts

**Figure 4:**
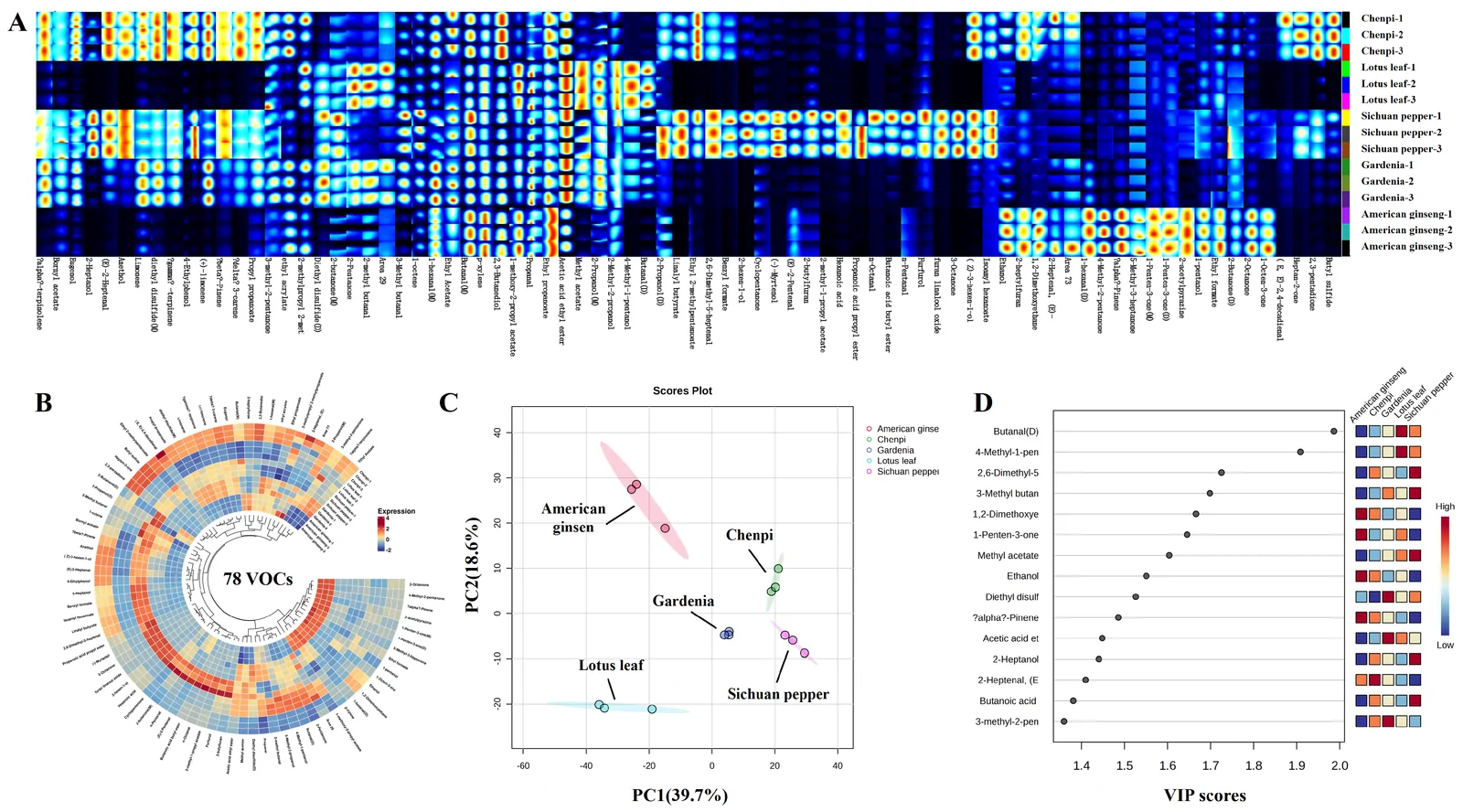
GC-IMS Analysis of Different MFH extracts (n = 3). GC-IMS fingerprints of MFH extracts (**A**). Ring-shaped heat map of GC-IMS (**B**). PCA of GC-IMS (**C**). VIP Scores of GC-IMS (**D**). Each point in (**C**) represents one analytical replicate.

A total of 78 volatile organic compounds (VOCs) were identified by GC-IMS, including aldehydes, ketones, alcohols, esters, acids, furans, and sulfur-containing compounds. As shown in Figure 4A, all MFH extracts exhibited distinct volatile fingerprints. However, substantial differences were observed in both signal intensities and volatile composition among the samples. Compounds such as Butanal, ethyl acetate, 2,3-butanediol, Ethyl propanoate, and 1-hexanal were commonly detected across all samples and were detected in all MFH extracts and were therefore considered commonly occurring VOC signals under the present analytical conditions. In contrast, D-limonene, α-pinene, 2-heptanone, and 1-octen-3-one were selectively enriched in specific samples, contributing to their unique aroma characteristics.

The circular heatmap (Figure 4B) and PCA (Figure 4C) consistently demonstrated that the five MFH extracts formed clearly separated volatile flavor profiles, indicating substantial differences in their botanical origin and aroma formation mechanisms. Furthermore, variable importance in projection (VIP) analysis (Figure 4D) identified butanal (D), 4-methyl-1-pentanol, 3-methylbutanal, α-pinene, and 2,6-dimethyl-5-heptenal as the most discriminative volatile markers. These compounds are primarily generated through lipid oxidation, amino acid degradation, and terpene metabolic pathways and are therefore considered important variables contributing to the statistical discrimination of the GC-IMS profiles.

Overall, GC-IMS proved to be an effective tool for characterizing the volatile flavor profiles of different medicinal and edible plant extracts and enabled a preliminary discrimination among various raw materials. However, relying solely on volatile compound composition for the identification of medicinal and edible materials from different sources still presents certain limitations as it cannot comprehensively reflect their overall flavor characteristics. In particular, GC-IMS is unable to directly quantify or evaluate the sensory perception experienced by consumers during consumption. Therefore, complementary evaluation techniques and multidimensional analytical approaches are required to further validate and supplement the findings, thereby providing a more comprehensive understanding of the flavor attributes of MFH materials.

### 3.3. Analysis of Cerebral Response Power Spectra to Different MFH Extracts

**Figure 5:**
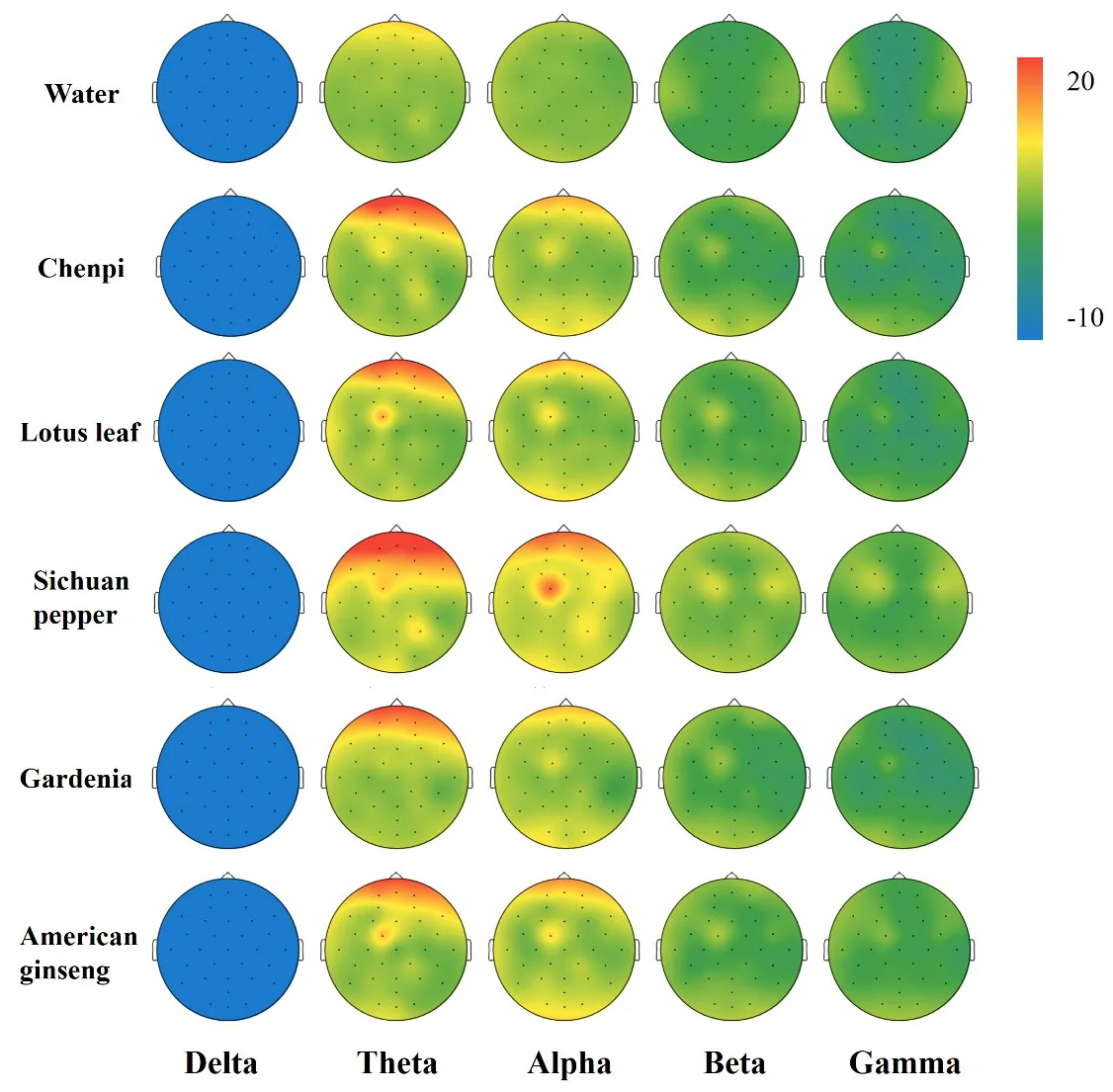
The average electroencephalogram topograms of the brain stimulated by different MFH extracts.

The temporal variations and geographic distributions of responses from the brain to taste stimuli were analyzed using time-evolving topographical maps of average EEG signal intensity [8,23]. Initially, the study evaluated the mean power of EEG responses to various MFH extracts over a 10 s sensory exposure. Figure 5 shows the average EEG topograms evoked by each MFH extract in the subject; color gradations from blue to red indicate increasing EEG power. Except in the delta band, different MFH extracts elicited markedly different activation patterns and intensities. Compared with the control (water group), different bitter stimuli have a greater activation degree on the brain’s theta, alpha, beta, and gamma frequency bands, especially the theta and alpha frequency bands, which correspond to 4–8 Hz and 8–14 Hz respectively. In the study of Tóth et al. [24], they investigated changes in electroencephalogram (EEG) activity in anorexia nervosa patients and healthy controls following sweet (milk chocolate) and bitter (black tea) taste stimuli by using electrophysiological methods. Results revealed that, compared to controls, anorexia patients exhibited a significantly higher percentage of theta wave power across all taste stimulus types and brain hemisphere sides.

Furthermore, it can be observed from the graph that the brain topograms corresponding to different stimuli are mainly distributed in the front of the brain, and they mainly correspond to the temporal and frontal regions of the human body. Previous studies indicated that the temporal cortex may be preferentially influenced by taste stimuli [25]. Yang et al. [26] demonstrated signal responses in the temporal lobe across various tastes, and found the main brain responsive regions involved in bitterness were the superior temporal gyrus, middle temporal gyrus, inferior temporal gyrusin, superior parietal lobule, inferior parietal lobule, inferior occipital gyrus, middle occipital gyrus, and the anterior- and mid-insula, which were similar to our research findings. At the same time, the anterior insula and frontal operculum make up the primary gustatory cortex, which handles taste perception [27].

### 3.4. Time-Frequency Examination of Brain Responses to Various Bitterness Stimuli

**Figure 6:**
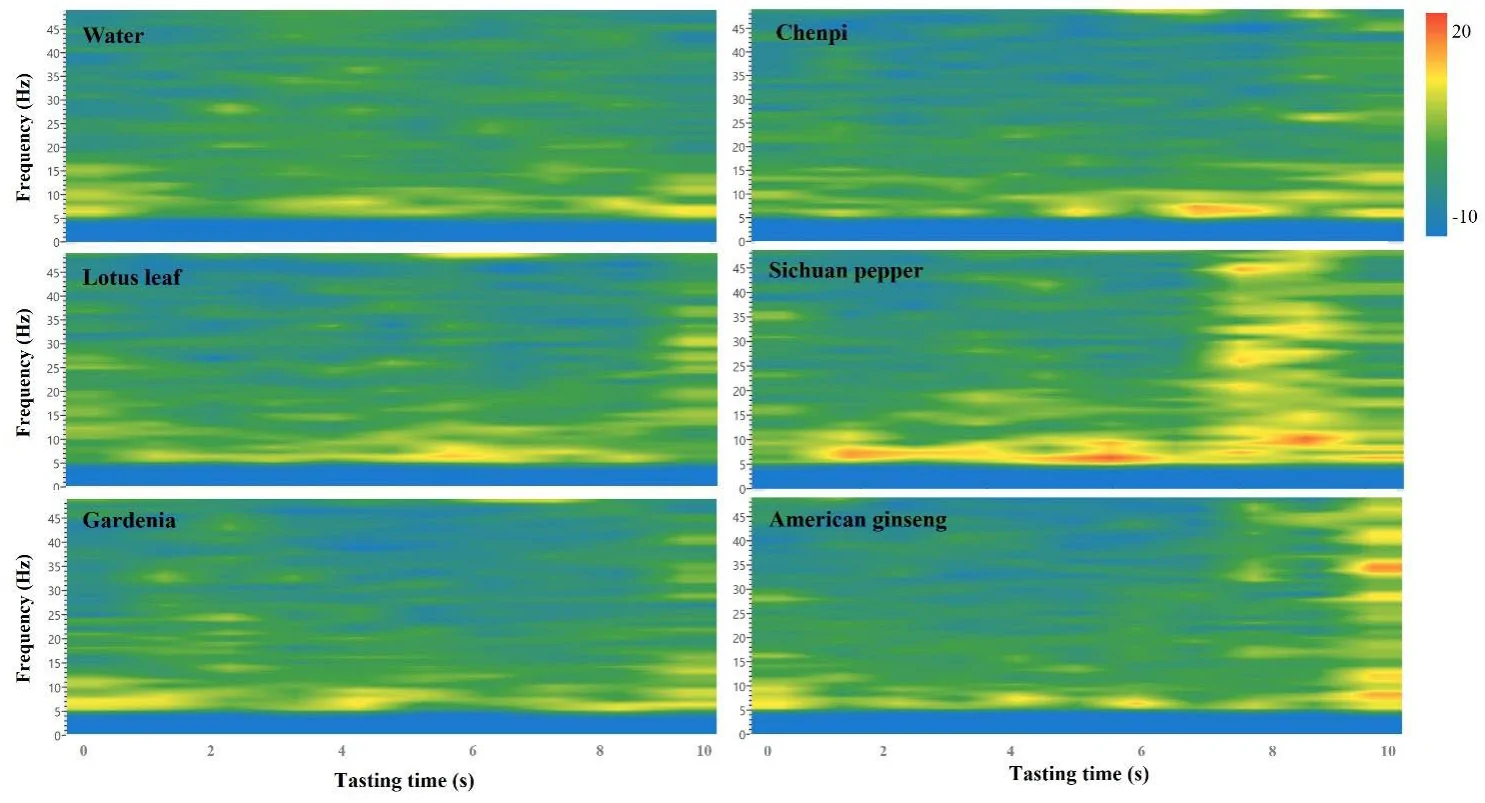
Time-Frequency maps of brain for differences between six taste stimuli.

We conducted Short-Time Fourier Transform (STFT) analysis to produce time-frequency spectrograms for each stimulus, thereby visualizing the dynamic spectrum alterations during the tasting period (0–10 s) (Figure 6). The color scale represents the power intensity, with red indicating high energy and blue indicating a low-energy baseline. As illustrated in Figure 6, the Water control group exhibited a stable, low-power baseline (predominantly blue/green) throughout the 10 s interval, indicating minimal cortical arousal. In contrast, the MFH extracts induced distinct spectral modulations. For Gardenia, Chenpi, and Lotus leaf, the induced spectral energy was primarily concentrated in the low-frequency bands (0–15 Hz, corresponding to δ, θ, α), appearing as intermittent bursts of activity (yellow/orange zones) rather than continuous sustainment. An apparent difference was observed for Sichuan pepper, which showed a delayed increase in spectral power beginning at approximately 6 s and continuing until the end of the recording period. This increase extended into the β and low-γ frequency ranges (>20 Hz). Alkylamides such as hydroxy-α-sanshool are known to produce tingling paresthesia involving trigeminal somatosensory pathways [28]. However, sanshool concentration and trigeminal activity were not directly measured in the present study, and the temporal correspondence alone does not establish a trigeminal origin of the observed EEG pattern. Moreover, subtle facial, lingual, or jaw-muscle activity associated with oral stimulation may have contributed to the high-frequency signal. Therefore, the delayed β–γ response represents a distinctive but exploratory scalp-EEG feature of Sichuan pepper stimulation, whose physiological origin remains to be established. Similarly, American ginseng displayed a time-dependent response, with peak energy concentrations emerging in the late phase (8–10 s, <15 Hz), potentially reflecting its characteristic “aftertaste” or lingering bitterness [29]. In summary, the time-frequency analysis reveals that while most bitter MFH extracts trigger low-frequency cortical synchronization, specific stimuli like Sichuan pepper induce high-arousal, broadband, and delayed neural responses, distinguishing them from pure taste perception [30].

### 3.5. Illustrative Scalp-Potential Patterns During Different Stimulation Conditions

**Figure 7:**
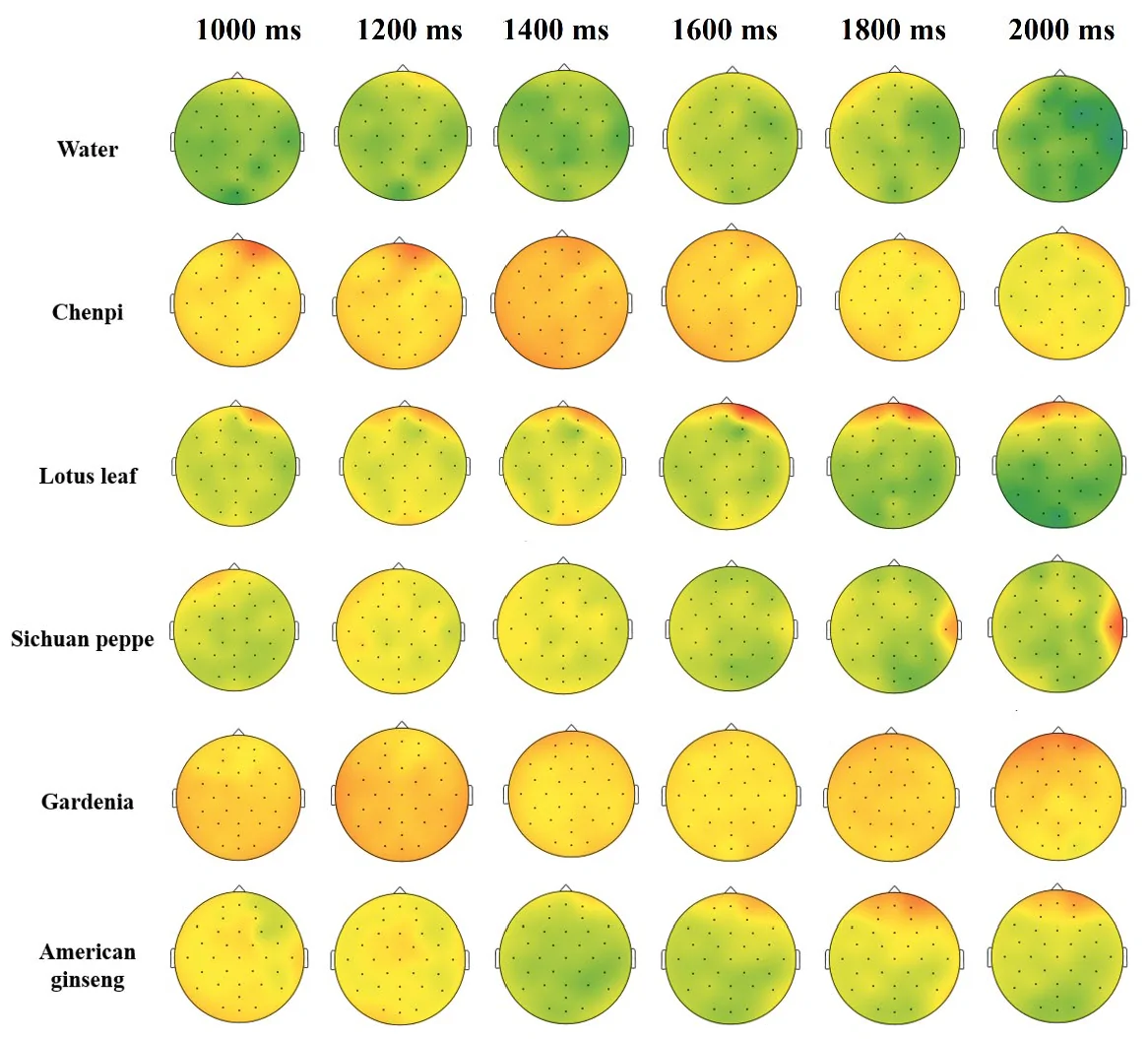
Illustrative scalp potential maps from one randomly selected participant during six stimulation conditions (Water, Chenpi, Lotus leaf, Sichuan pepper, Gardenia, and American ginseng). Color coding is relative to the absolute maximum potential difference. The position of all 32 channels is marked with black dots.

Figure 7 presents illustrative scalp-potential maps from one randomly selected participant under the six stimulation conditions. In this participant, the spatial distribution and magnitude of scalp potentials varied visually across conditions and time points. The water-control condition showed relatively small scalp-potential variations throughout the observation window, with only minor fluctuations over the frontal scalp area. Under the Gardenia condition, relatively higher and more broadly distributed scalp-potential magnitudes, represented by the orange-red areas, were visually observed across much of the 1000–2000 ms interval. The Chenpi condition also showed broadly distributed scalp-potential changes, although their visual magnitude appeared lower than that observed under the Gardenia condition. Under the Lotus leaf condition, the scalp-potential changes appeared initially over the frontal scalp area and became more pronounced at later time points, particularly at approximately 1800–2000 ms. Under the Sichuan pepper condition, relatively small scalp-potential changes were observed at the earlier time points, followed by a localized increase over the right temporal-parietal scalp area from approximately 1600 ms onward. Overall, Figure 7 illustrates stimulus- and time-related variations in scalp-potential patterns in one participant; however, in the absence of supporting evidence from group-level comparisons, cortical source localization, or direct assessments of specific sensory-processing mechanisms, these patterns should be interpreted as descriptive and participant-specific.

### 3.6. The Power of Theta Band of Regional Brain Responses to Various Stimulations

**Figure 8:**
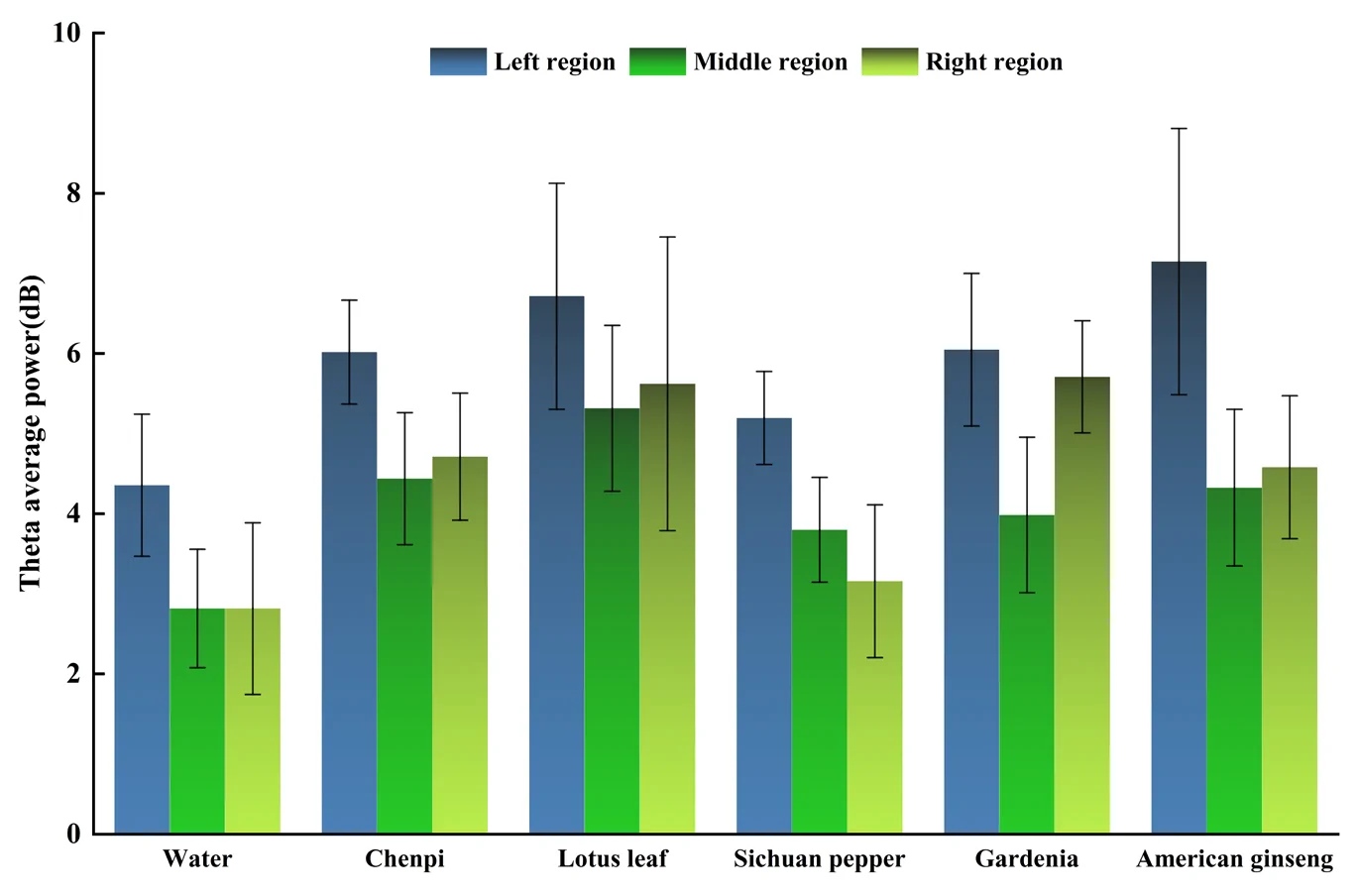
Regional EEG power responses to the different MFH stimuli. Data are presented as mean ± SD. Differences were evaluated using a two-factor repeated-measures ANOVA, with stimulus type and brain region as within-subject factors and participant as the repeated-measures subject.

To characterize the scalp-recorded EEG responses associated with different MFH extracts, we calculated the average PSD power (dB) within the theta band (4–8 Hz; Figure 8). A two-factor repeated-measures ANOVA revealed significant main effects of stimulus, F(2.27, 9.09) = 19.52, Greenhouse-Geisser-adjusted *p* < 0.001, ηp^2^ = 0.830, and scalp region, F(1.06, 4.23) = 22.34, adjusted *p* = 0.008, ηp^2^ = 0.848. Holm-adjusted comparisons of the marginal regional means showed that theta power was significantly higher in the left region than in the middle and right regions (adjusted *p* = 0.017 and 0.028, respectively). However, the stimulus × region interaction was not significant, F(2.55, 10.19) = 1.11, adjusted *p* = 0.382, ηp^2^ = 0.217, indicating that the regional distribution of theta power did not differ significantly among the stimulation conditions. Collectively, the regional analysis demonstrated a consistent predominance of theta-band power over the left scalp region across the MFH stimulation conditions, with significantly higher marginal power than that observed over the middle and right regions.

Theta oscillations are believed to be closely related to emotional processing, attention regulation, and the integration of sensory information. In taste research, the activity in the theta frequency band often occurs during the processing of aversive stimuli (such as bitterness), reflecting the brain’s alertness and avoidance mechanisms for potentially harmful substances [26]. Bitterness, as a congenital aversive taste, can rapidly activate taste centers, including the anterior insula, orbitofrontal cortex, and anterior cingulate gyrus, all of which are closely related to the generation of theta oscillations [31]. The results from this study indicated that the theta frequency band has a significant feedback response to bitter taste perception, and the left brain region has a significantly higher response intensity than the central and right brain regions. Nevertheless, the present regional differences cannot be assigned specifically to bitterness because the extracts also differed in aroma, astringency, pungency, numbing sensation, color, and mouthfeel. These results therefore provide exploratory evidence of regional scalp EEG variation during complex MFH stimulation rather than proof of a bitterness-processing mechanism or hemispheric dominance.

### 3.7. Comparison of Electrode Differences Related to Bitterness Among Different Raw Materials

Table 1 presents the variations in EEG signal magnitudes recorded at specific electrodes (Fz, F3, F4, F7, F8, AF3, AF4, FC1, FC2, FC5, FC6, T7, and T8) following the ingestion of extracts from various MFH extracts as well as a water group. As illustrated in the table, the recorded values across the specified electrodes generally ranged between 13 and 14 dB. Notably, significantly higher values (mean ~14 dB) were consistently observed at the F7 (left frontal) and T7 (left temporal) electrodes across all stimuli, including the water control. This suggests that these brain regions exhibit the highest level of activation during the processing of liquid stimuli, potentially reflecting a generalized response to gustatory or mouthfeel stimulation. Conversely, the AF3 and AF4 (prefrontal) and F4 (right frontal) electrodes exhibited generally lower values (mean range: 10–11 dB), indicating a relatively attenuated level of activation in these regions. Furthermore, the data reveal that the EEG response patterns for Chenpi were most similar to those of water, suggesting that the cortical processing of its bitterness is relatively mild. In contrast, American ginseng displayed a greater degree of data dispersion (indicated by larger standard deviations). This points to significant inter-subject variability in the perception of its bitterness, a finding that aligns with sensory evaluation results where subjective bitterness perception of American ginseng varied markedly among participants.

### 3.8. Inter-Channel Correlations of Scalp EEG Power

**Figure 9:**
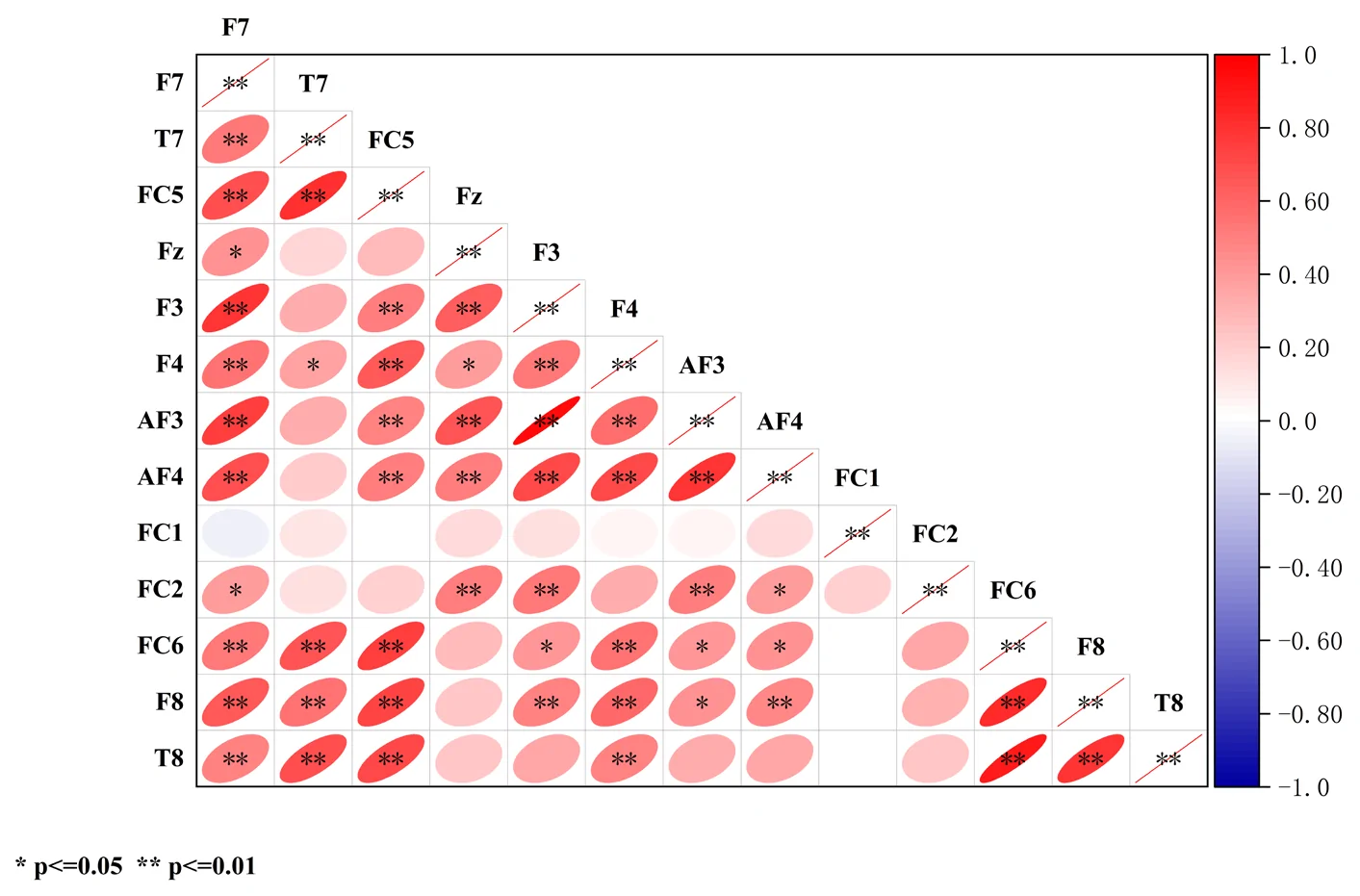
Pearson correlation matrix of EEG power among selected scalp electrodes during MFH extract stimulation. Ellipse color and orientation represent the direction and magnitude of the correlation coefficient.

To examine the associations among electrode-level EEG responses during MFH extract stimulation, Pearson correlation analysis was performed using the power measurements from 13 selected scalp electrodes (F7, T7, FC5, Fz, F3, F4, AF3, AF4, FC1, FC2, FC6, F8, and T8). As shown in Figure 9, most electrode pairs exhibited positive correlations, although their magnitudes and statistical significance varied.

Relatively strong positive correlations were observed among the left lateral electrodes F7, T7, and FC5. A similar pattern was found among the right lateral electrodes FC6, F8, and T8, particularly for FC6-F8, FC6-T8, and F8-T8. Several frontal and prefrontal electrode pairs, including F3-AF3, F3-AF4, AF3-AF4, and F3-F4, also showed significant positive correlations. In addition, F7 and FC5 were positively correlated with several frontal and contralateral electrodes, indicating that similar changes in scalp EEG power occurred across multiple recording sites during the stimulation conditions. In contrast, FC1 showed generally weak or nonsignificant correlations with most other electrodes. These findings describe the co-variation of EEG power recorded at different scalp electrodes during MFH stimulation [26]. The correlations between frontal, frontocentral, and temporal/lateral electrodes may reflect broadly distributed scalp-level response patterns associated with the complex oral sensory characteristics of the extracts.

Previous studies have established that the insular cortex (primary gustatory cortex), located deep within the lateral sulcus, works in conjunction with the prefrontal cortex (secondary gustatory cortex) to process taste intensity and hedonic value (unpleasantness) [32]. The high correlation between T7/T8 (temporal) and AF3/AF4 (prefrontal) observed here likely reflects the correlated scalp-level activity patterns between the sensory identification of bitterness and the subsequent cognitive or emotional evaluation (e.g., aversion) of the bitter stimulus [33].

Undoubtedly, our research is limited by various constraints. Taste perception is multifaceted and influenced by sensory, emotional, and hedonic expectations, whereas this study focused primarily on EEG responses to MFH extracts without directly evaluating emotional responses [34]. The relatively small sample size and absence of an a priori power analysis may have limited the detection of small effects and complex interactions. Handedness and familiarity with the tested products were not recorded, restricting the interpretation of apparent lateralization. Moreover, the extracts differed not only in bitterness but also in aroma, astringency, pungency, mouthfeel, and numbing sensation; therefore, the EEG differences cannot be attributed exclusively to bitterness. Trigeminal activity and sanshool concentrations were not directly measured, and residual oral-muscle activity may have contributed to the β–γ signals observed during Sichuan pepper stimulation. The regional electrode groupings were partly data-informed and should be considered exploratory, while scalp EEG cannot reliably localize deep gustatory structures. Finally, GC-IMS, E-nose, and E-tongue provided complementary flavor profiles but did not establish causal compound–perception–neural relationships. Larger, prospectively powered studies using predefined regions, standardized stimuli, improved artifact control, and direct chemical and trigeminal measurements are needed to validate these findings.

## 4. Conclusions

This study provided an exploratory multimethod characterization of bitterness-related sensory, chemical, and scalp-EEG responses to selected MFH extracts. A key finding was the observed mismatch between sensor-derived bitterness response and human sensory perception, particularly in Sichuan pepper, which exhibited strong bitterness signals in the E-tongue but relatively low perceived bitterness. EEG analysis revealed an apparent delayed increase in β–γ power during Sichuan pepper stimulation, representing a distinctive exploratory scalp-EEG pattern whose physiological origin remains uncertain because potential contributions from oral stimulation-related myogenic activity cannot be excluded. In contrast, conventional bitter stimuli such as Gardenia primarily evoked low-frequency neural oscillations associated with typical bitter perception.

Taken together, GC-IMS, sensory evaluation, E-tongue, and EEG provided complementary characterization of the chemical, perceptual, and scalp-electrophysiological responses elicited by the different MFH extracts. The observed differences suggest that perceived bitterness may be influenced by multiple sensory attributes, including gustatory, olfactory, and trigeminal cues. Furthermore, the inter-channel correlations observed between frontal and temporal electrodes may reflect scalp-level activity patterns potentially associated with sensory discrimination and affective evaluation during bitterness perception. The proposed multimethod characterization offers a promising approach for objective flavor evaluation and provides theoretical support for the development of targeted bitterness-masking strategies and consumer-oriented MFH products.

## Figures and Tables

**Table 1:** Descriptive electrode-level EEG values under the six stimulation conditions.

	Fz	F3	F4	F7	F8	AF3	AF4	FC1	FC2	FC5	FC6	T7	T8
Water	12.31 ± 1.76 ^bc^	12.42 ± 1.27 ^bc^	10.95 ± 1.81 ^de^	14.19 ± 1.61 ^a^	11.77 ± 2.5 ^bcde^	10.87 ± 1.60 ^e^	11.17 ± 2.32 ^cde^	12.21 ± 3.44 ^bcd^	11.69 ± 2.02 ^bcde^	11.79 ± 1.76 ^bcde^	11.63 ± 2.3 ^bcde^	14.19 ± 2.32 ^a^	12.89 ± 3.49 ^b^
Chenpi	12.60 ± 2.47 ^b^	12.17 ± 1.04 ^bcd^	10.97 ± 2.43 ^cd^	14.44 ± 2.39 ^a^	11.3 ± 2.11 ^bcd^	10.79 ± 1.56 ^d^	11.3 ± 2.55 ^bcd^	12.43 ± 4.62 ^bcd^	11.61 ± 2.20 ^bcd^	11.77 ± 1.59 ^bcd^	11.75 ± 2.01 ^bcd^	13.96 ± 2.26 ^a^	12.15 ± 3.01 ^bcd^
Lotus leaf	12.92 ± 3.28 ^bc^	12.95 ± 3.93 ^bc^	10.69 ± 2.01 ^d^	14.68 ± 2.46 ^a^	11.57 ± 2.79 ^cd^	11.45 ± 4.24 ^cd^	11.16 ± 2.57 ^cd^	11.58 ± 2.75 ^cd^	11.82 ± 2.76 ^cd^	11.93 ± 2.16 ^cd^	12.13 ± 2.98 ^cd^	14.49 ± 3.18 ^ab^	12.60 ± 3.80 ^c^
Sichuan pepper	13.02 ± 3.18 ^bc^	12.40 ± 1.62 ^cd^	11.17 ± 2.08 ^d^	14.74 ± 2.29 ^a^	11.72 ± 3.01 ^cd^	11.15 ± 2.23 ^d^	11.83 ± 2.48 ^cd^	13.21 ± 5.34 ^bc^	11.97 ± 2.90 ^cd^	12.04 ± 1.73 ^cd^	11.97 ± 1.99 ^cd^	13.99 ± 2.31 ^ab^	13.14 ± 3.34 ^bc^
Gardenia	12.51 ± 2.38 ^b^	12.46 ± 1.62 ^b^	11.28 ± 2.64 ^bc^	14.36 ± 2.25 ^a^	12.13 ± 3.49 ^bc^	10.79 ± 1.50 ^c^	11.51 ± 2.22 ^bc^	11.96 ± 3.42 ^bc^	11.83 ± 3.54 ^bc^	11.75 ± 1.87 ^bc^	11.70 ± 2.00 ^bc^	14.06 ± 2.39 ^a^	12.44 ± 3.12 ^b^
American ginseng	13.67 ± 4.03 ^ab^	13.17 ± 3.93 ^abc^	10.71 ± 1.93 ^e^	14.63 ± 2.49 ^a^	11.7 ± 2.54 ^cde^	11.41 ± 4.06 ^cde^	11.05 ± 2.36 ^de^	11.36 ± 2.77 ^de^	11.69 ± 2.41 ^cde^	11.89 ± 2.50 ^cde^	11.96 ± 2.90 ^cde^	14.28 ± 2.82 ^ab^	12.78 ± 3.89 ^bcd^

Note: Values are presented as mean ± standard deviation. Different lowercase letters indicate significant differences among electrode values within the same stimulus (*p* < 0.05).

## Data Availability

The original contributions presented in the study are included in the article/Supplementary Materials, further inquiries can be directed to the corresponding author.

## Supplementary Materials

The following supporting information can be downloaded at: https://www.mdpi.com/article/10.3390/foods15183181/s1, Table S1: Detailed information regarding the different MFH materials. Table S2: Physicochemical properties of the five MFH extracts. Table S3: Information on volatile compounds identified in different MFH samples by GC–IMS.
