# Supplementary material for: Decoding Bitter Taste Perception in Medicine-Food Homology Substances: A Multimethod Data and Sensory Assessment Approach
Source: Foods. 2026 Sep 9;15(18):3181. doi: 10.3390/foods15183181 (PMC13605653; doi:10.3390/foods15183181)
Supplement: Supplementary file 1 [file foods-15-03181-s001.zip › foods-4480644-supplementary.pdf]

Table S1 Detailed information regarding the different MFH materials

Common name	Botanical name	Family	Plant part	Origin	Batch No.
Chenpi	*Citrus reticulata Blanco*	Rutaceae	Mature dried pericarp	Quanzhou, Fujian Province, China	Not available
Lotus leaf	*Nelumbo nucifera Gaertn.*	Nelumbonaceae	Dried leaf	Sichuan Province, China	Not available
Sichuan pepper	*Zanthoxylum bungeanum Maxim.*	Rutaceae	Dried pericarp	Sichuan Province, China	Not available
Gardenia	*Gardenia jasminoides J.Ellis*	Rubiaceae	Dried ripe fruit	Jiangxi Province, China	Not available
American ginseng	*Panax quinquefolius L.*	Araliaceae	Dried root	Jilin Province, China	Not available

Table S2 Physicochemical properties of the five MFH extracts

Sample name	pH	Total soluble solids (°Brix)	Turbidity (NTU)
Chenpi	4.35 ± 0.08^{d}	2.33 ± 0.06^{b}	161.33 ± 2.08^{c}
Lotus leaf	5.17 ± 0.05^{b}	1.23 ± 0.06^{c}	159.00 ± 1.00^{c}
Sichuan pepper	4.20 ± 0.02^{e}	1.27 ± 0.12^{c}	552.67 ± 1.53^{b}
Gardenia	4.48 ± 0.12^{c}	2.50 ± 0.30^{b}	1514.67 ± 14.01^{a}
American ginseng	5.46 ± 0.02^{a}	2.80 ± 0.10^{a}	152.33 ± 0.58^{c}

Note: Values are expressed as mean ± SD (n = 3). Total soluble solids are expressed as °Brix and turbidity as NTU. Different superscript letters within the same column indicate significant differences among samples (one-way ANOVA followed by Tukey's test, $P < 0.05$).

Table S3 Information on volatile compounds identified in different MFH samples by GC–IMS

Count	Compound	CAS#	Formula	MW	RI	Rt [sec]	Dt
1	(E, E)-2,4-decadienal	C25152-84-5	$C_{10}H_{16}O$	152.2	1740.4	1632.964	1.4317
2	furan linalool oxide	C60047-17-8	$C_{10}H_{18}O_2$	170.3	1486.9	964.11	1.25095
3	2-heptylfuran	C3777-71-7	$C_{11}H_{18}O$	166.3	1402	808.233	1.40469
4	(Z)-3-hexen-1-ol	C928-96-1	$C_6H_{12}O$	100.2	1359.1	739.146	1.21488
5	2,6-Dimethyl-5-heptenal	C106-72-9	$C_9H_{16}O$	140.2	1357.2	736.369	1.70078
6	2-hexen-1-ol	C2305-21-7	$C_6H_{12}O$	100.2	1391.7	791.065	1.1895
7	2-Heptanol	C543-49-7	$C_7H_{16}O$	116.2	1324.5	687.918	1.72702
8	Isoamyl hexanoate	C2198-61-0	$C_{11}H_{22}O_2$	186.3	1266.9	619.165	2.13514
9	(-)-Myrtenol	C19894-97-4	$C_{10}H_{16}O$	152.2	1179.7	541.871	2.17183
10	(E)-2-Heptenal (D)	C18829-55-5	$C_7H_{12}O$	112.2	1311.1	669.04	1.65465
11	3-Octanone	C106-68-3	$C_8H_{16}O$	128.2	1293.4	644.856	1.72023
12	gamma -terpinene	C99-85-4	$C_{10}H_{16}$	136.2	1265.3	617.682	1.70131
13	Delta-3-carene	C13466-78-9	$C_{10}H_{16}$	136.2	1174.8	537.793	1.63321
14	alpha-terpinolene	C586-62-9	$C_{10}H_{16}$	136.2	1323.5	686.431	1.21699
15	diethyl disulfide(M)	C110-81-6	$C_4H_{10}S_2$	122.2	1265	617.411	1.28258
16	n-Octanal	C124-13-0	$C_8H_{16}O$	128.2	1251.6	604.911	1.8123
17	Heptan-2-one	C110-43-0	$C_7H_{14}O$	114.2	1232.9	587.792	1.71266
18	Bornyl acetate	C76-49-3	$C_{12}H_{20}O_2$	196.3	1311.9	670.127	1.21573
19	Limonene	C138-86-3	$C_{10}H_{16}$	136.2	1267.9	620.128	1.21195
20	Anethol	C104-46-1	$C_{10}H_{12}O$	148.2	1291.5	642.953	1.21195
21	Butanoic acid butyl ester	C109-21-7	$C_8H_{16}O_2$	144.2	1251.6	604.857	1.33556
22	Butyl sulfide	C544-40-1	$C_8H_{18}S$	146.3	1232.2	587.149	1.2937
23	4-Ethylphenol	C123-07-9	$C_8H_{10}O$	122.2	1233.4	588.241	1.20189
24	(+)-limonene	C138-86-3	$C_{10}H_{16}$	136.2	1175.7	538.574	1.21842
25	Ethyl 2-methylpentanoate	C39255-32-8	$C_8H_{16}O_2$	144.2	1148.8	516.847	1.29362
26	2-butylfuran	C4466-24-4	$C_8H_{12}O$	124.2	1115.2	490.926	1.16722
27	1-hexanal(M)	C66-25-1	$C_6H_{12}O$	100.2	1107	484.827	1.26642
28	1-hexanal(D)	C66-25-1	$C_6H_{12}O$	100.2	1107.3	485.018	1.56241
29	2-methylpropyl 2-methylpropanoate	C97-85-8	$C_8H_{16}O_2$	144.2	1066.7	437.37	1.81201
30	Propanoic acid propyl ester	C106-36-5	$C_6H_{12}O_2$	116.2	1078.2	452.045	1.20722
31	(E)-2-Pentenal	C1576-87-0	C_5H_8O	84.1	1112.1	488.639	1.36722
32	Benzyl formate	C104-57-4	$C_8H_8O_2$	136.1	1051.4	418.501	1.44241
33	2-methyl-1-propyl acetate	C110-19-0	$C_6H_{12}O_2$	116.2	1029.3	392.771	1.60561
34	2,3-pentadione	C600-14-6	$C_5H_8O_2$	100.1	1038.7	403.444	1.28882

Count	Compound	CAS#	Formula	MW	RI	Rt [sec]	Dt
35	2-acetylpyrazine	C22047-25-2	$C_6H_6N_2O$	122.1	1046.3	412.402	1.13202
36	4-Methyl-2-pentanone	C108-10-1	$C_6H_{12}O$	100.2	1029	392.409	1.18039
37	Hexanoic acid	C142-62-1	$C_6H_{12}O_2$	116.2	1029	392.445	1.29189
38	ethyl acrylate	C140-88-5	$C_5H_8O_2$	100.1	1003.6	364.79	1.42371
39	(E)-2-Heptenal (M)	C18829-55-5	$C_7H_{12}O$	112.2	934.2	304.25	1.67501
40	2-methyl butanal	C96-17-3	$C_5H_{10}O$	86.1	945.2	313.068	1.40099
41	3-methyl-2-pentanone	C565-61-7	$C_6H_{12}O$	100.2	1004.8	365.981	1.18518
42	Acetic acid ethyl ester	C141-78-6	$C_4H_8O_2$	88.1	918.1	291.856	1.33426
43	2-Pentanone	C107-87-9	$C_5H_{10}O$	86.1	945.3	313.191	1.36341
44	2-butanone(M)	C78-93-3	C_4H_8O	72.1	948.1	315.458	1.26163
45	2-Propanol(M)	C67-63-0	C_3H_8O	60.1	934.7	304.708	1.09634
46	2-Methyl-2-propanol	C75-65-0	$C_4H_{10}O$	74.1	943.6	311.822	1.32078
47	2-Propanol(D)	C67-63-0	C_3H_8O	60.1	946.6	314.246	1.22074
48	Diethyl disulfide(D)	C110-81-6	$C_4H_{10}S_2$	122.2	933.9	304.082	1.28164
49	2-Butanone(D)	C78-93-3	C_4H_8O	72.1	933.1	303.418	1.24162
50	1,2-Dimethoxyethane	C110-71-4	$C_4H_{10}O_2$	90.1	923	295.6	1.112
51	Ethyl propanoate	C105-37-3	$C_5H_{10}O_2$	102.1	956.2	322.103	1.13984
52	n-Pentanal	C110-62-3	$C_5H_{10}O$	86.1	975	338.146	1.18982
53	Propyl propanoate	C106-36-5	$C_6H_{12}O_2$	116.2	1039.1	403.996	1.21575
54	Ethyl Acetate	C141-78-6	$C_4H_8O_2$	88.1	914.2	288.955	1.09502
55	1-octene	C111-66-0	C_8H_{16}	112.2	881.8	265.689	1.15804
56	3-Methyl butanal	C590-86-3	$C_5H_{10}O$	86.1	884.4	267.479	1.19433
57	Butanal(M)	C123-72-8	C_4H_8O	72.1	867.3	255.927	1.28027
58	Methyl acetate	C79-20-9	$C_3H_6O_2$	74.1	869.4	257.31	1.19433
59	Butanal(D)	C123-72-8	C_4H_8O	72.1	876.1	261.784	1.11221
60	p-xylene	C106-42-3	C_8H_{10}	106.2	860.4	251.392	1.06919
61	Propanal	C123-38-6	C_3H_6O	58.1	817.3	224.843	1.13522
62	2,3-Butanediol	C513-85-9	$C_4H_{10}O_2$	90.1	813.4	222.609	1.35146
63	Ethyl formate	C109-94-4	$C_3H_6O_2$	74.1	795.7	212.62	1.06401
64	4-Methyl-1-pentanol	C626-89-1	$C_6H_{14}O$	102.2	857.8	249.683	1.32038
65	Furfurol	C98-01-1	$C_5H_4O_2$	96.1	844.3	241.14	1.08602
66	1-methoxy-2-propyl acetate	C108-65-6	$C_6H_{12}O_3$	132.2	860	251.129	1.14558
67	Eugenol	C97-53-0	$C_{10}H_{12}O_2$	164.2	1316.4	676.42	1.29878
68	2-Octanone	C111-13-7	$C_8H_{16}O$	128.2	1302.6	657.37	1.33785
69	1-Octen-3-one	C4312-99-6	$C_8H_{14}O$	126.2	1302.9	657.679	1.69006
70	1-pentanol	C71-41-0	$C_5H_{12}O$	88.1	1298.6	651.817	1.25972
71	beta-Pinene	C127-91-3	$C_{10}H_{16}$	136.2	1146.1	514.741	1.21214
72	Linalyl butyrate	C78-36-4	$C_{14}H_{24}O_2$	224.3	1392.1	791.659	1.21725
73	alpha-Pinene	C80-56-8	$C_{10}H_{16}$	136.2	1003.1	364.202	1.29067
74	Cyclopentanone	C120-92-3	C_5H_8O	84.1	1210.7	568.199	1.11634
75	Ethanol	C64-17-5	C_2H_6O	46.1	910.6	286.252	1.12711

Count	Compound	CAS#	Formula	MW	RI	Rt [sec]	Dt
76	5-Methyl-3-heptanone	C541-85-5	$C_8H_{16}O$	128.2	914.4	289.065	1.27839
77	1-Penten-3-one(M)	C1629-58-9	C_5H_8O	84.1	1032.9	396.819	1.0902
78	1-Penten-3-one(D)	C1629-58-9	C_5H_8O	84.1	1024.2	386.984	1.09057
